# Cancer survival differentials for Aboriginal and Torres Strait Islander peoples in Queensland: the impact of remoteness

**DOI:** 10.1007/s10552-022-01643-1

**Published:** 2022-10-20

**Authors:** S. M. Cramb, L. J. Whop, G. Garvey, P. D. Baade

**Affiliations:** 1 Australian Centre for Health Services Innovation & Centre for Healthcare Transformation, Queensland University of Technology, Brisbane, QLD Australia; 2 School of Public Health and Social Work, Queensland University of Technology, Brisbane, QLD Australia; 3 Centre for Data Science, Queensland University of Technology, Brisbane, QLD Australia; 4 National Centre for Epidemiology and Population Health, Australian National University, Canberra, ACT Australia; 5 School of Public Health, Faculty of Medicine, The University of Queensland, Brisbane, QLD Australia; 6 Cancer Council Queensland, Brisbane, QLD Australia; 7 School of Mathematical Sciences, Queensland University of Technology, Brisbane, QLD Australia; 8 Menzies Institute of Health Research, Griffith University, Gold Coast, QLD Australia

**Keywords:** Aboriginal and Torres Strait Islander, Health inequity, Flexible parametric survival, Cancer, Australia

## Abstract

**Purpose:**

In Australia, Aboriginal and Torres Strait Islander peoples (First Nations population) often have low overall cancer survival, as do all residents of geographically remote areas. This study aimed to quantify the survival disparity between First Nations and other Queenslanders for 12 common cancer types by remoteness areas.

**Methods:**

For all Queensland residents aged 20–89 years diagnosed with a primary invasive cancer during 1997–2016, we ran flexible parametric survival models incorporating age, First Nations status, sex, diagnosis time period, area-level socioeconomic status, remoteness categories and where appropriate, broad cancer type. Three survival measures were predicted: cause-specific survival, survival differences and the comparative survival ratio, each standardised to First Nations peoples’ covariate distributions.

**Results:**

The standardised five-year cause-specific cancer survival was 60% for urban First Nations and 65% for other Queenslanders, while remote residents were 54% (First Nations) and 58% (other). The absolute survival differential between First Nations and other Queenslanders was often similar, regardless of remoteness of residence. The greatest absolute difference in five-year standardised cancer survival was for head and neck cancers, followed by cervical cancer.

The five-year comparative survival ratio (First Nations: other Queenslanders) for urban cancer patients was 0.91 (95% CI 0.90–0.93), similar to outer regional, inner regional and remote areas. The greatest comparative survival differential was for oesophageal cancer.

**Conclusion:**

First Nations’ survival inequalities are largely independent of geographical remoteness. It remains a priority to determine the contribution of other potential factors such as the availability of culturally acceptable diagnostic, management and/or support services.

**Supplementary Information:**

The online version contains supplementary material available at 10.1007/s10552-022-01643-1.

## Introduction

The poorer cancer survival experienced by Aboriginal and Torres Strait Islanders, the First Nations peoples of Australia, is well-documented [1–8]. Australians residing in remote geographical areas also experience lower survival from cancer than those living in more urban areas [9]. Reasons proposed for these poorer cancer survival outcomes include diminished access to cancer diagnostic, treatment [10] and primary health-care services [11], as well as generally lower socioeconomic conditions [12].

To date, there has been limited examination of whether the extent of lower survival experienced by First Nations peoples diagnosed with cancer varies by geographic remoteness. Previous Australian studies looking at the survival differential by geographical areas have considered either deaths from any cause among cancer patients [1], or have only reported survival among First Nations peoples for all cancer types combined [13].

The First Nations population represents around 3.3% of the total Australian population, with Queensland having the second largest First Nations population (4.6%) after New South Wales [14]. Within Queensland, almost half of the First Nations population resides in outer regional or remote/very remote areas [14]. We aimed to quantify the extent of the survival disparity faced by First Nations people in Queensland across geographical remoteness for commonly diagnosed cancer types.

## Methods

Data on all First Nations peoples and other Queensland residents aged 20–89 years diagnosed with a primary invasive cancer between 1997 and 2016 were obtained from the Queensland Cancer Register following ethics approval from the Metro South Health Human Research Ethics Committee (HREC/2019QMS/57005) and the data custodians. This population-based cancer registry receives notification of all cancers diagnosed (except for keratinocyte cancers) among Queensland residents. Cases were followed up to 31 December 2016 by routine matching to the National Death Index.

In recent years the Queensland Cancer Register has implemented what is known as a Multi-Stage Median (MSM) algorithm to identify First Nations people among notified cases [15]. The MSM combines data relating to First Nations status from multiple data sources such as public and private hospitals, nursing homes and death certificates. This new method provides a more complete estimation of First Nations status compared to what has been used previously[16].

Specific cancer types were included if at least 100 cases were diagnosed and more than 50 cancer-specific deaths among First Nations people in the study cohort. If multiple primary cancers were diagnosed, only the first primary cancer diagnosis was included. Time between diagnosis and death (or censoring) was measured in days. Cases were censored at whichever came first: 10 years after diagnosis, 31 December 2016 or if they died from a cause other than the diagnosed cancer, at the date of death. Cases were excluded if First Nations ethnicity was missing (0.83%), residential information was missing (0.84%), were diagnosed by autopsy (0.13%) or death certificate (0.79%) or survived for less than one day (0.14%).

Residential information was provided at the Statistical Area level 2 (SA2) level, with boundaries defined on the 2011 Australian Statistical Geography Standard [17]. SA2s vary greatly in land size, and in 2016 had a median estimated resident population of 8,341 people (90% interval: 2,581–19,002) [18]. In 2011, there were 526 Queensland SA2s with a physical location, with 11,036 smaller Statistical Areas level 1 (SA1s) nested within [17]. Remoteness categories [19], which are defined based on relative access to services within each SA1, were allocated to each SA2 based on the remoteness category having the highest population within that SA2, using an official concordance [20]. Four levels of remoteness were considered: Urban (corresponds to Major City), Inner regional, Outer regional and Remote (combines Remote and Very Remote). Each SA2 was assigned to the appropriate Socio-Economic Indexes for Areas using the Index of Relative Socioeconomic Advantage and Disadvantage (IRSAD) for Queensland in 2011 [21], and three categories were formed: Disadvantaged (lowest 20%), Average (middle 60%) and Advantaged (highest 20%). People whose SA2 did not have an IRSAD decile were also removed (0.01%).

Survival analyses used flexible parametric survival models [22, 23], which estimate the baseline survival function using restricted cubic splines to enable greater flexibility in shape. The final model was based on the hazard scale and included age as a nonlinear continuous variable using restricted cubic splines, First Nations status (yes/no), sex (males/females), year of diagnosis (1997–2006; 2007–2016), area-level socioeconomic status (disadvantaged/average/advantaged) and remoteness categories (urban/inner regional/outer regional/remote). The effect of each covariate was allowed to vary with follow-up time if proportional hazards assumptions were not met. The number of knots selected for the age splines, baseline complexity and time-varying effects for each cancer type is based on both Bayesian Information Criterion values, plots of martingale residuals and parsimony. To account for the possibility that the distribution of cancer types was different between First Nations and Other Queenslanders, the models for aggregated cancer groups (all cancers combined; head and neck cancers) were additionally adjusted for broad cancer site groups based on the Queensland five-year cause-specific survival among persons for individual cancer types in 1997–2016 and collapsed into four groups (0– < 25%,25– < 50%,50–< 75%,75–100%).

Each flexible parametric model checked for two-way interactions between remoteness categories and First Nations ethnicity. The significance of the interaction terms was assessed using likelihood ratio tests and visual plots of the predicted values. Only significant interaction terms were retained in the final models for each cancer type.

These flexible parametric survival models were used to predict three measures that quantify the cause-specific survival disparity between First Nations and other Queenslanders diagnosed with cancer. All survival estimates were standardised by applying the age, sex and remoteness distribution of the First Nations cohort to the cohort for other Queenslanders. The three measures were considered for up to 10 years after diagnosis (1) *standardised cause-specific survival*; (2) *standardised cause-specific survival differences* which quantify the absolute differences between the standardised survival estimates for First Nations and other Queenslanders; and (3) *standardised comparative survival ratio* which quantifies the ratio of the standardised cause-specific survival estimates for First Nations people to that for other Queenslanders. A standardised comparative survival ratio of less than one indicates that survival among First Nations cancer patients is poorer than for other cancer patients [24].

Calculations were performed in Stata MP v16.0 (StataCorp, Texas) using the meansurv postestimation option for the stpm2 package [23]. Each of the survival measures described above was predicted from the flexible parametric survival models separately for First Nations peoples and other Queenslanders, stratified by remoteness of residence. Stata syntax for each measure is provided in Supplementary Material.

## Results

The final cohort comprised 5,791 First Nations peoples (1.5%) and 368,089 other Queensland residents diagnosed with cancer (Table 1). The median age at diagnosis was lower for First Nations people than other Queenslanders for all cancer types (Table 1). For First Nations people, diagnosed cancers were fairly equally distributed across remoteness categories, while for other Queenslanders most cancers occurred in urban residents; this was consistent with the population distribution for each group (Supplementary Table S1). Cancer proportions by area-level socioeconomic status also differed by ethnicity (Supplementary Table S2).

**Table 1:** Demographic characteristics of the study cohort, Queensland, 1997–2016

Type of cancer (ICD-10)	First Nations			Other Queenslanders		
	Cases	% all cancers	Median age	Cases	% all cancers	Median age
All cancers (C00–C97)	5,791	100	58	368,089	100	65
Head and neck cancers (C00–14, C30–32)	356	6	53	10,319	3	62
Oesophageal cancer (C15)	136	2	58.5	3,786	1	69
Stomach cancer (C16)	113	2	63	5,644	2	70
Colorectal cancer (C18–20, C218)	514	9	60.5	45,687	12	69
Pancreatic cancer (C25)	137	2	62	3,382	1	66
Liver cancer (C22)	137	2	62	7,036	2	70
Lung cancer (C33–34)	852	15	62	31,138	8	69
Breast cancer (C50)	734	13	54	48,306	13	59
Cervical cancer (C53)	203	4	43	3,044	1	45
Prostate cancer (C61)	129	2	54	9,822	3	67
Leukaemia (C91–95)	356	6	53	10,319	3	62

Breast cancer only includes females. Further details on demographic characteristics by remoteness and area-level socioeconomic status are available in Supplementary Tables S1–S2

In addition to all cancers combined, cancer types with significant evidence of interaction between remoteness and First Nations status were oesophageal, colorectal and pancreatic cancers and leukaemia. All cancer types had various time-varying effects included, except for cervical and head and neck cancers, as preferred by model diagnostics (Table 2).

**Table 2:** Final model specifications by type of cancer

	Number of knots specified			Variables included as time-varying effects	Main effect interactions
	Age spline terms	Baseline hazard	Time-varying effects		
All cancers	2	4	2	Age spline terms, broad cancer groups	Ethnicity × remoteness
Head and neck cancers	2	3	n.a	-	
Oesophageal cancer	3	2	1	Age spline terms	Ethnicity × remoteness
Stomach cancer	3	3	1	Age spline terms, sex, remoteness	
Colorectal cancer	3	5	3	Age spline terms, sex, broad time periods	Ethnicity × remoteness
Liver cancer	2	2	1	Age spline terms, broad time periods	
Pancreatic cancer	3	5	2	Age spline terms	Ethnicity × remoteness
Lung cancer	3	5	1	Age spline terms, remoteness	
Breast cancer	2	3	1	Age spline terms, remoteness, socioeconomic status	
Cervical cancer	2	3	n.a	-	
Prostate cancer	2	1	1	Age spline terms	
Leukaemia	3	5	3	Age spline terms, sex	Ethnicity × remoteness

Only ethnicity by remoteness was considered as a potential interaction term

### Standardised survival estimates

**Fig. 1:**
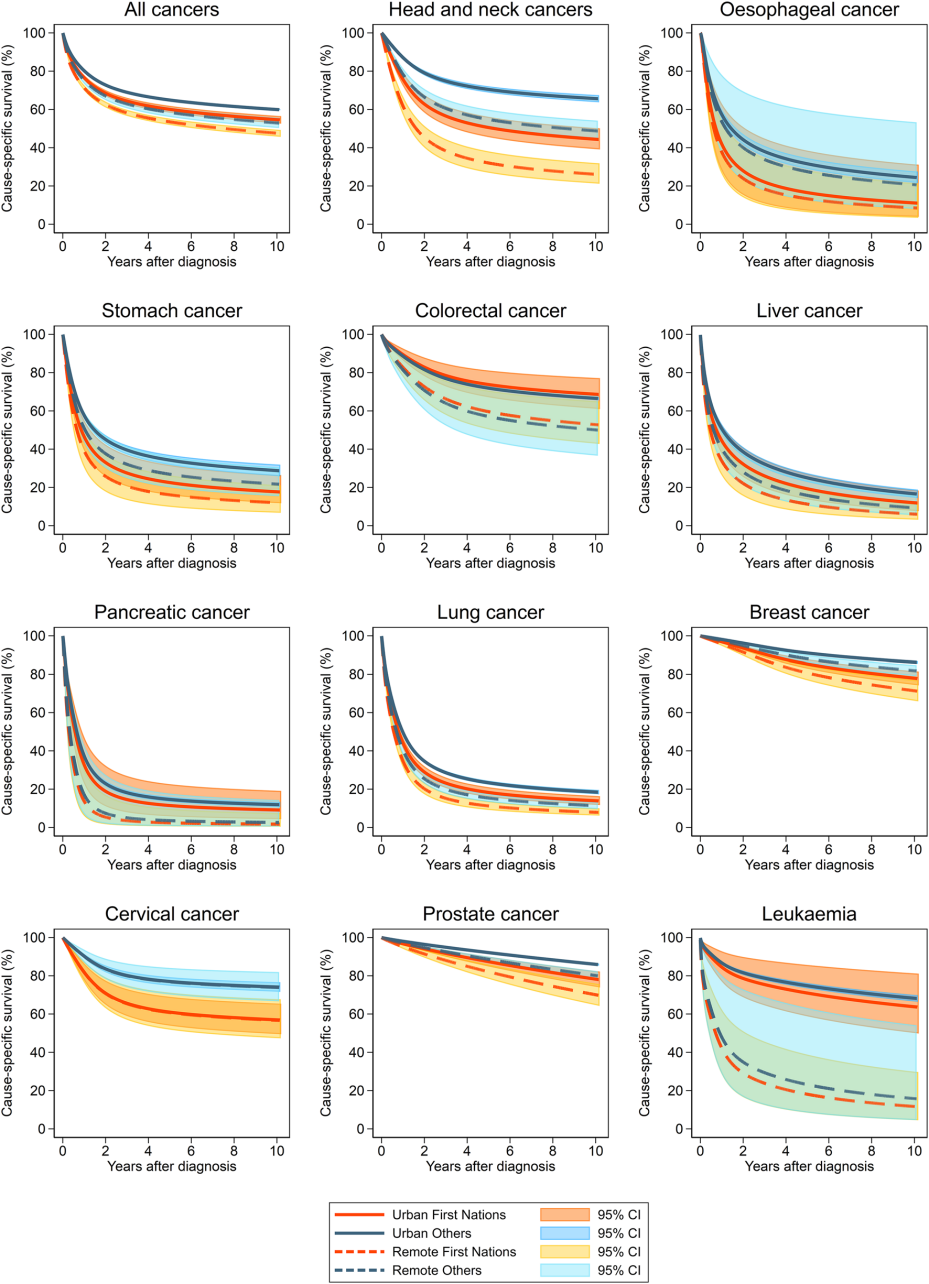
Standardised survival estimates for First Nations peoples and other Queenslanders for urban (= major city) and remote (= remote and very remote) areas of Queensland, 1997–2016. Other population standardised to the First Nations population characteristics. Breast cancer is for females only. Green shaded areas represent overlapping 95% CIs

Within a week from diagnosis, the predicted survival for First Nations and other Queenslanders was significantly different for both urban and remote residents (Fig. 1). By one-year after diagnosis, 77% of First Nations Queenslanders residing in urban areas had survived their cancer, compared with 80% of other urban residents (Table 3). Similar differentials were observed for remote residents, with First Nations cancer patients having a one-year cause-specific survival of 71% compared with 75% for other cancer patients. By five-years, among urban residents this had decreased to 60% and 65% for First Nations and other respectively, and among remote residents it was 54% (First Nations) and 58% (other).

**Table 3:** Standardised one and five-year cause-specific survival estimates by ethnicity and remoteness, Queensland, 1997–2016

Cancer	One-year standardised survival (95% CI)				Five-year standardised survival (95% CI)			
	Urban		Remote		Urban		Remote	
	First Nations	Other Queenslanders	First Nations	Other Queenslanders	First Nations	Other Queenslanders	First Nations	Other Queenslanders
All cancers	76.5 (75.1, 77.9)	80.2 (80.0, 80.4)	71.2 (69.8, 72.6)	75.3 (73.4, 77.3)	60.0 (58.3, 61.9)	64.9 (64.7, 65.1)	53.5 (51.9, 55.2)	58.5 (56.1, 61.0)
Head and neck cancers	75.7 (72.3, 79.3)	87.1 (86.2, 88.0)	61.7 (57.0, 66.8)	78.4 (75.4, 81.4)	50.5 (45.5, 56.1)	70.4 (68.8, 72.1)	32.0 (26.9, 38.1)	54.8 (50.1, 59.8)
Oesophageal cancer	41.9 (27.8, 63.1)	57.3 (54.8, 60.0)	37.7 (26.3, 54.2)	53.6 (36.8, 78.1)	16.5 (7.1, 38.5)	31.6 (28.9, 34.6)	13.3 (6.3, 28.2)	27.5 (12.6, 59.7)
Stomach cancer	46.4 (39.1, 55.1)	57.6 (55.7, 59.5)	39.1 (30.2, 50.6)	50.8 (43.4, 59.5)	22.5 (16.0, 31.7)	34.2 (31.7, 37.0)	16.1 (9.9, 26.3)	26.9 (20.0, 36.1)
Colorectal cancer	89.4 (86.4, 92.6)	88.6 (88.2, 88.9)	82.7 (77.7, 88.0)	81.4 (74.2, 89.3)	73.8 (67.1, 81.1)	71.9 (71.2, 72.6)	59.6 (50.3, 70.6)	57.1 (44.3, 73.4)
Liver cancer	44.4 (37.4, 52.7)	50.6 (48.3, 53.1)	33.8 (26.3, 43.5)	40.2 (32.7, 49.4)	19.3 (13.7, 27.1)	25.0 (22.8, 27.4)	11.3 (6.8, 18.6)	15.8 (10.5, 23.8)
Pancreatic cancer	32.7 (22.6, 47.1)	37.2 (35.6, 38.9)	13.6 (6.0, 30.8)	17.0 (6.7, 42.9)	11.4 (5.8, 22.6)	14.6 (13.3, 16.0)	2.4 (0.6, 9.7)	3.6 (0.7, 17.7)
Lung cancer	44.0 (41.2, 47.0)	49.6 (48.7, 50.5)	34.7 (31.3, 38.4)	40.4 (37.6, 43.4)	18.3 (15.9, 21.0)	23.3 (22.3, 24.4)	11.2 (9.1, 13.8)	15.3 (13.3, 17.6)
Breast cancer	96.8 (96.3, 97.5)	98.1 (98.0, 98.3)	95.7 (94.7, 96.8)	97.5 (97.0, 98.0)	85.3 (82.9, 87.8)	91.0 (90.6, 91.5)	80.7 (76.8, 84.7)	88.0 (86.1, 90.1)
Cervical cancer	81.8 (77.5, 86.4)	90.2 (89.0, 91.5)	81.7 (76.2, 87.5)	90.1 (86.8, 93.6)	61.0 (53.9, 69.0)	77.0 (74.8, 79.3)	60.7 (51.8, 71.2)	76.8 (70.2, 84.1)
Prostate cancer	96.7 (96.0, 97.5)	98.0 (97.9, 98.1)	95.2 (94.0, 96.4)	97.1 (96.7, 97.5)	87.4 (84.8, 90.0)	92.1 (91.8, 92.4)	82.1 (78.4, 85.9)	88.6 (87.1, 90.0)
Leukaemia	84.5 (77.0, 92.8)	86.7 (85.8, 87.6)	42.2 (28.5, 62.4)	47.9 (28.6, 80.1)	71.0 (58.8, 85.6)	74.8 (73.4, 76.2)	18.1 (8.4, 38.8)	23.1 (8.5, 63.1)

Urban = Major City Remoteness Areas; Remote = combined Remote and Very Remote Remoteness Areas. For 10 year survival estimates, refer to Supplementary Table S3

Survival varied by cancer type, with the highest five-year cancer survival among First Nations urban residents observed for prostate cancer (87%) and the lowest for pancreatic cancer (11%) (Fig. 1; Table 3). The same ranking was observed among First Nations residents of remote areas (prostate cancer 82%; pancreatic cancer 2%).

The greatest remoteness differential among First Nations cancer patients was observed for leukaemia (five-year urban survival 71% versus remote 18%), followed by head and neck cancers (urban five-year survival 51% versus 32% remote) (Table 3; Fig. 1; Supplementary Fig. S1).

While urban First Nations residents often had similar survival to remote other Queenslanders, cervical cancer had lower survival (urban First Nations five-year survival: 61%, remote other Queenslanders: 77%) (Table 3). Breast cancer survival also showed this pattern, although with a smaller differential.

### Standardised survival differences

**Fig. 2:**
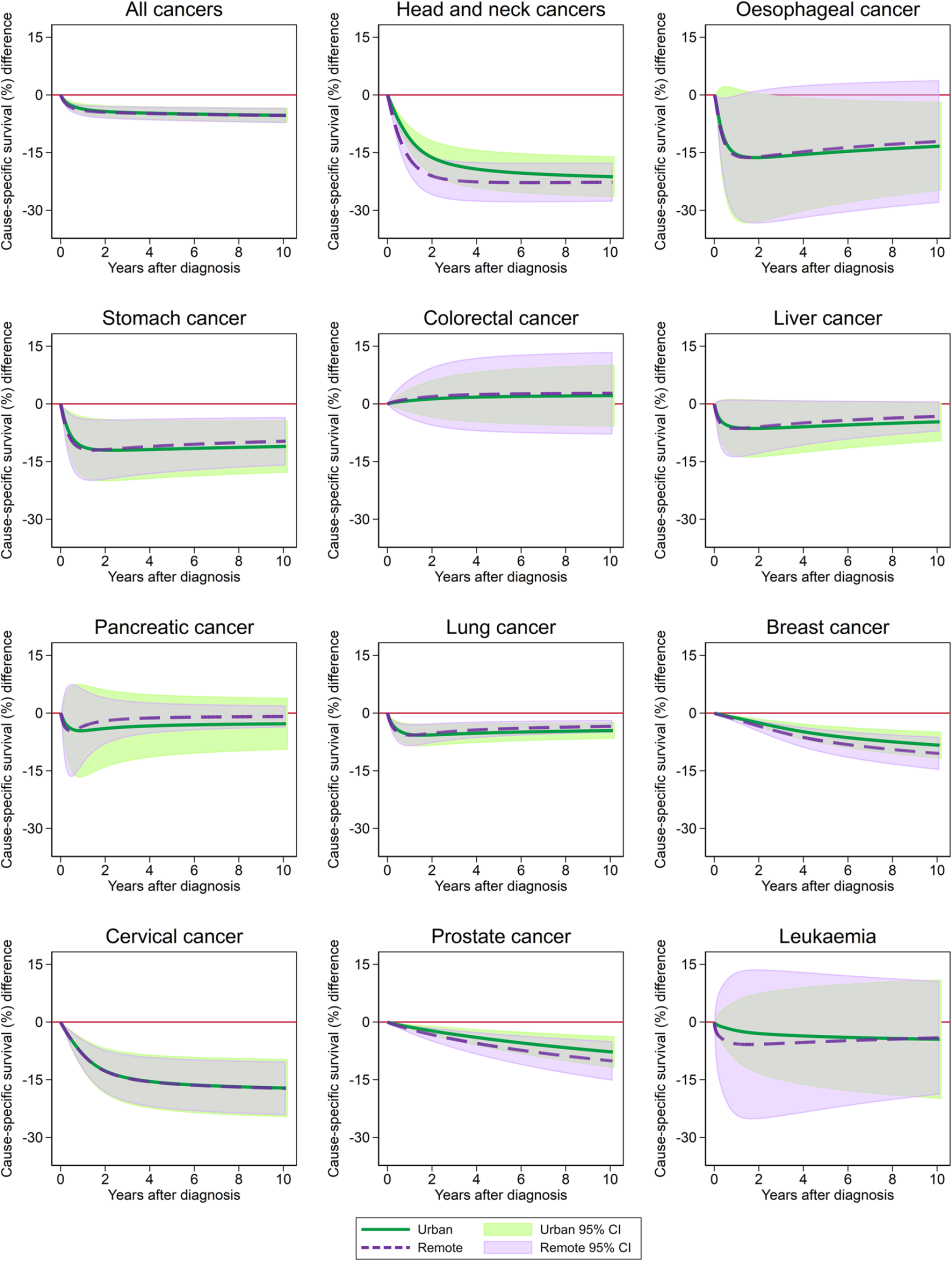
Standardised survival differences between First Nations peoples and other Queenslanders for urban (= major city) and remote (= remote and very remote) areas of Queensland, 1997–2016. Other population standardised to the First Nations population characteristics. Breast cancer is for females only. Grey shaded areas represent overlapping 95% CIs

For all cancers combined, the difference in survival rates between First Nations and other Queenslanders rapidly increased within the first year from diagnosis among both urban and remote residents and then remained fairly consistent at around 5% lower survival among First Nations cancer patients regardless of location (Fig. 2; Supplementary Fig. S2).

While the magnitude varied, the consistency of this differential by location held across each cancer type examined (Fig. 2). Although some cancers suggested greater differences among remote residents, this was never significant.

Breast and prostate cancers tended to show increasing differences in cancer survival (i.e., moving away from zero) between First Nations and other Queenslanders as time from diagnosis increased (Fig. 2).

Head and neck cancers showed the largest absolute survival difference (~ 20% at 5 years), and this increased slightly by 10 years (Fig. 2). Cervical cancer also had large discrepancies in survival (16–17%) between First Nations and other Queenslanders across the full 10 years.

For the other cancer types examined, the survival disparity decreased (i.e., approached zero) with increasing time from diagnosis, approaching no differences by 10 years from diagnosis (Fig. 2; Supplementary Fig. S2). This was true both for cancer types with lower survival (lung, liver, pancreatic) and higher survival (leukaemia).

### Comparative survival ratio

**Fig. 3:**
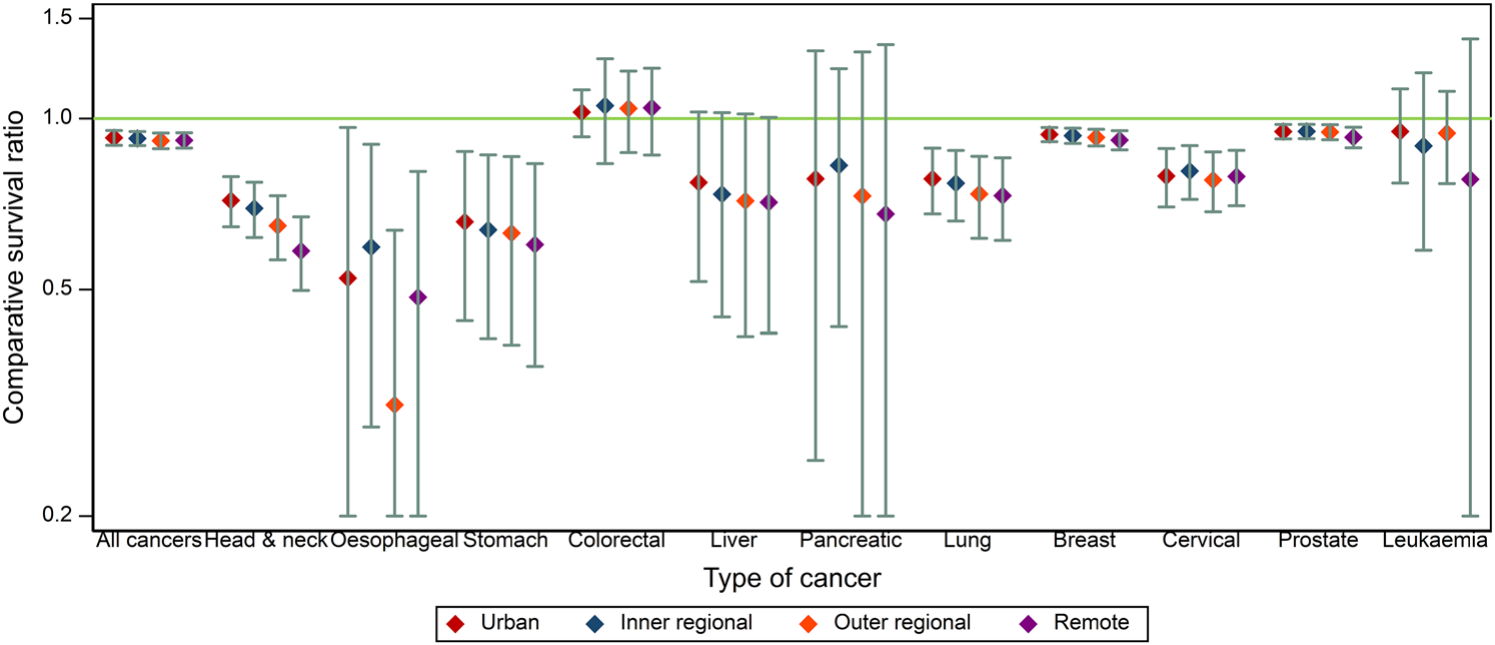
Five-year comparative survival ratios by cancer type and remoteness, Queensland, 1997–2016. The comparative survival ratio is the standardised cause-specific five-year survival for First Nations people divided by other Queenslanders. A value of 1 means First Nations survival up to five-years is equivalent to other Queenslander survival. Capped lines show the 95% CI. Urban is the Major City remoteness area. Remote is the combined Remote and Very Remote remoteness areas. Breast cancer is for females only. Refer to Supplementary Fig. S3 for 10 year estimates

The comparative survival ratio for First Nations cancer patients living in urban areas surviving five-years was 0.93 (95% CI 0.90–0.95), meaning they had significantly lower survival than other Queenslanders (Fig. 3). This was similar to that obtained for outer regional, inner regional and remote areas. Certain cancers had markedly greater comparative survival differentials, with oesophageal cancer having the greatest across all areas (urban: 0.52, inner regional: 0.59, outer regional: 0.32, remote: 0.48), however there was large uncertainty around these. The next greatest in urban areas was stomach cancer (0.66) and in remote areas was head and neck cancers (0.58). These rankings remained the same at 10 years, with slightly greater comparative differentials (Supplementary Fig. S3).

## Discussion

First Nations cancer patients faced a consistently poorer survival outcome than other Queenslanders for most cancer types regardless of their location, with little evidence of remoteness influencing the magnitude of the survival differential faced by First Nations cancer patients. For several key cancer types the survival gap between First Nations and other Queenslanders diminished with time from diagnosis, highlighting the importance of the time period immediately following diagnosis.

The lower survival for all Australian cancer patients living in more remote areas, irrespective of whether they were First Nations peoples or not, is consistent with the vast distances and small populations in these communities, causing difficulties in ensuring sufficient coverage of health services [25]. This has been associated with later diagnosis of cancer [26], less treatment [27] and/or lack of access to clinical trials [28]. Digital interventions such as telemedicine are increasingly being considered as an adjunct service [29, 30], and have already demonstrated benefit in rural Aboriginal health-care settings [31].

However, the lack of evidence for interaction between remoteness and First Nations status for many cancer types suggests that remoteness itself is not driving the cancer survival differential between First Nations peoples and other Queenslanders. A recent study [32] reported that there remained a significant survival differential for First Nations cancer patients in Queensland even after adjusting for remoteness of residence, consistent with our findings of survival differences across all remoteness categories. While this pattern does vary by cancer type, it suggests that cultural acceptability of current diagnostic and treatment services may be more of an issue than the availability of services determined by geographic location.

There are complex barriers to First Nations people accessing appropriate and timely cancer care. Cultural safety is a key concern of First Nations people interacting with the health system in Australia [33, 34]. Some options with demonstrated potential include providing First Nations care coordinators [34] or patient navigators [35]. More First Nations health workers and/or enhanced reach and coverage of Aboriginal primary health services would also help [36]. Better understanding the enablers and barriers for First Nations people to participate in cancer screening plus access and complete treatment has been identified as a research priority [37].

The pattern of lower survival among urban First Nations than remote other Queenslanders is most pronounced among those diagnosed with breast and cervical cancers. Although cervical cancer is one of the most preventable cancer types, the impact of HPV vaccinations would not have been observed in our study cohort [38], and pap smear cervical screening has lower First Nations participation across all areas of Queensland [39, 40]. In contrast, breast cancer, when detected early and treated appropriately, has very high survival rates, yet mammogram participation among First Nations women is low [41]. This lower survival for breast and cervical cancer is consistent with the lower respective screening participation, which in turn suggest that current screening services are not equally accessible for First Nations women. Increasing cancer screening programs’ cultural safety is critical to addressing survival inequities. The introduction of innovative new technology, such as the HPV self-collection method that will be universally available in Australia from 1 July 2022, holds great promise since it has been shown to be acceptable and increase cervical screening for under screened populations [42, 43].

Oesophageal cancer and head and neck cancers both had consistently very low survival among First Nations cancer patients in comparison to other Queenslanders, across all remoteness levels. While there is little information on causes specific to low survival from oesophageal cancer, poorer First Nations’ survival from head and neck cancer was attributed to receiving less treatment [44]. Investing in and expanding First Nations’ programs aimed at reducing the incidence and mortality for these cancers is vital [45].

The results presented demonstrate the survival disparities if the other Queensland population had the same age structure as the First Nations population, so that the reported differences are independent of any age differences. Younger people tend to have higher survival from cancer [46], but although First Nations Queenslanders are diagnosed at younger ages, they generally have lower survival. This standardisation by age aimed to then demonstrate the true magnitude of differences.

Limitations of our study included the lack of information on cancer stage and treatment received. There is some evidence to suggests First Nations cancer patients in urban areas may be diagnosed at more advanced cancer stages, have more comorbidities and are less likely to receive treatment [8, 47], but cancer stage, treatment and comorbidities are not collected by the Queensland Cancer Register, so our data preclude exploring these very influential factors. Socioeconomic status is often influential on cancer survival, but we could only incorporate an area-level, rather than individual-level, measure. Finally, it remains possible that the lack of evidence for interaction between remoteness and First Nations ethnicity for some cancer types is due to the small numbers of cases rather than necessarily a true absence of interaction.

Advantages of using these flexible parametric models include the greater ease of including time-varying components, continuous variables such as for age, and interaction terms. While our modelled estimates are dependent on the model specifications, our sensitivity analyses indicated that these models fit the data well and were not sensitive to the number of knots used in the spline terms. Other study strengths include using data from the Queensland Cancer Register, which is known to have high quality First Nations ethnicity information available since 1997, and practically complete coverage of all cancers.

Given that a recent Queensland study [32] has shown little change in the survival disparity across all the state over the last two decades, it is vital to understand the underlying causes of these survival inequalities. Our results provide evidence that the survival inequalities are independent of geographical remoteness. It remains a priority to determine the contribution of other factors such as the availability of culturally acceptable diagnostic, management and/or support services.

## Supplementary Information

Below is the link to the electronic supplementary material.

Supplementary file1 (DOCX 780 kb)

## Data Availability

The dataset analysed during the current study requires approval from the data custodians following ethics approval. The modelled survival estimates can be obtained from the corresponding author on reasonable request.

## References

[1] Australian Institute of Health and Welfare, Cancer Australia (2013) Cancer in Aboriginal and Torres Strait Islander peoples of Australia: an overview. Cancer series 78. Cat. no. CAN 75. AIHW, Canberra

[2] Cramb SM, Garvey G, Valery PC, Williamson JD, Baade PD (2012). The first year counts: cancer survival among Indigenous and non-Indigenous Queenslanders, 1997–2006. Med J Aust.

[3] Condon JR, Zhang X, Baade P, Griffiths K, Cunningham J, Roder DM (2014). Cancer survival for Aboriginal and Torres Strait Islander Australians: a national study of survival rates and excess mortality. Popul Health Metrics.

[4] Bernardes CM, Diaz A, Valery PC, Sabesan S, Baxi S, Aoun S (2019). Unmet supportive care needs among indigenous cancer patients across Australia. Rural Remote Health..

[5] Dasgupta P, Baade PD, Youlden DR, Garvey G, Aitken JF, Wallington I (2017). Variations in outcomes for Indigenous women with breast cancer in Australia: a systematic review. Eur J Cancer Care (Engl).

[6] Diaz A, Moore SP, Martin JH, Green AC, Garvey G, Valery PC (2015). Factors associated with cancer-specific and overall survival among Indigenous and non-Indigenous gynecologic cancer patients in Queensland, Australia: a matched cohort study. Int J Gynecol Cancer.

[7] Moore SP, Green AC, Bray F, Coory M, Garvey G, Sabesan S (2016). Colorectal cancer among Indigenous and non-Indigenous people in Queensland, Australia: toward survival equality. Asia Pac J Clin Oncol.

[8] Moore SP, Green AC, Bray F, Garvey G, Coory M, Martin J (2014). Survival disparities in Australia: an analysis of patterns of care and comorbidities among Indigenous and non-Indigenous cancer patients. BMC Cancer.

[9] Australian Institute of Health and Welfare (2012) Cancer survival and prevalence in Australia: period estimates from 1982 to 2010. Cancer Series no. 69. Cat. no. CAN 65. AIHW, Canberra 10.1111/ajco.12062 23418847

[10] Afshar N, English DR, Milne RL (2019). Rural-urban residence and cancer survival in high-income countries: a systematic review. Cancer.

[11] Australian Institute of Health and Welfare (2019) Cancer in Australia. Cancer series no.119. Cat. no. CAN 123. AIHW, Canberra

[12] Afshar N, English DR, Chamberlain JA, Blakely T, Thursfield V, Farrugia H (2020). Differences in cancer survival by remoteness of residence: an analysis of data from a population-based cancer registry. Cancer Causes Control.

[13] Tervonen HE, Aranda S, Roder D, Walton R, Baker D, You H (2016). Differences in impact of Aboriginal and Torres Strait Islander status on cancer stage and survival by level of socio-economic disadvantage and remoteness of residence - a population-based cohort study in Australia. Cancer Epidemiol.

[14] Australian Bureau of Statistics (2018) Estimates of Aboriginal and Torres Strait Islander Australians, June 2016. ABS, Canberra

[15] Christensen D, Davis G, Draper G, Mitrou F, McKeown S, Lawrence D (2014). Evidence for the use of an algorithm in resolving inconsistent and missing Indigenous status in administrative data collections. Aust J Soc Issues.

[16] Dunn N, Mitchell L, Cossio D. Asking the question - missing Indigenous identification underestimates the impact of different cancers. Australasian Epidemiological Association Annual Scientific Meeting; Brisbane. 2019

[17] Australian Bureau of Statistics (2011) Australian Statistical Geography Standard (ASGS): Volume 1 – Main structure and greater capital city statistical areas, 2011. ABS, Canberra

[18] Australian Bureau of Statistics (2019) ABS.Stat Dataset: ERP by Statistical Areas Level 2 (SA2) geographical areas (ASGS 2011), Age and Sex, 2001 to 2016. ABS Cat No 3235.0. ABS, Canberra

[19] Australian Bureau of Statistics (2013) Australian Statistical Geography Standard (ASGS): Volume 5 – Remoteness Structure, July 2011 ABS, Canberra

[20] Australian Bureau of Statistics (2012) 1270.0.55.006 – Australian Statistical Geography Standard (ASGS): Correspondences, July 2011: Statistical Area Level 2 2011 to Remoteness Area 2011. ABS, Canberra

[21] Australian Bureau of Statistics (2013) Census of Population and Housing: Socio-Economic Indexes for Areas (SEIFA), Australia, 2011. ABS, Canberra

[22] Royston P, Lambert PC (2011). Flexible parametric analysis using Stata: beyond the Cox model.

[23] Lambert PC, Royston P (2009). Further development of flexible parametric models for survival analysis. Stata Journal.

[24] Baade PD, Dasgupta P, Dickman PW, Cramb S, Williamson JD, Condon JR (2016). Quantifying the changes in survival inequality for Indigenous people diagnosed with cancer in Queensland. Australia Cancer Epidemiol.

[25] Barreto SG (2020). Pancreatic cancer in Australia: is not it time we address the inequitable resource problem?. Future Oncol.

[26] Baade PD, Dasgupta P, Aitken J, Turrell G (2011). Geographic remoteness and risk of advanced colorectal cancer at diagnosis in Queensland: a multilevel study. Br J Cancer.

[27] Depczynski J, Dobbins T, Armstrong B, Lower T (2019). Comparative use of cancer therapies in Australian farm, rural nonfarm and urban residents aged 45 years and older. Public Health Res Pract.

[28] Muthusamy A, Long D, Underhill CR (2021). Improving recruitment to clinical trials for regional and rural cancer patients through a regionally based clinical trials network. Med J Aust.

[29] Rollin A, Ridout B, Campbell A (2018). Digital health in melanoma posttreatment care in rural and remote Australia: systematic review. J Med Internet Res.

[30] Paterson C, Bacon R, Dwyer R, Morrison KS, Toohey K, O'Dea A (2020). The role of telehealth during the COVID-19 pandemic across the interdisciplinary cancer team: implications for practice. Semin Oncol Nurs.

[31] Smith AC, Armfield NR, Caffery LJ (2019). Telehealth a game changer: closing the gap in remote Aboriginal communities. Med J Aust.

[32] Peng Y, Baade P (2021). Survival disparities among recently diagnosed Aboriginal and Torres Strait Islander cancer patients in Australia remain. Cancer Causes Control.

[33] Green M, Anderson K, Griffiths K, Garvey G, Cunningham J (2018). Understanding Indigenous Australians’ experiences of cancer care: stakeholders’ views on what to measure and how to measure it. BMC Health Serv Res.

[34] Reilly R, Micklem J, Yerrell P, Banham D, Morey K, Stajic J (2018). Aboriginal experiences of cancer and care coordination: lessons from the cancer data and Aboriginal disparities (CanDAD) narratives. Health Expect.

[35] Bernardes CM, Martin J, Cole P, Kitchener T, Cowburn G, Garvey G (2018). Lessons learned from a pilot study of an Indigenous patient navigator intervention in Queensland. Australia.

[36] Australian Institute of Health and Welfare (2020) Aboriginal and Torres Strait Islander Health Performance Framework 2020 summary report. Cat. no. IHPF 2. AIHW, Canberra

[37] Morris B, Anderson K, Cunningham J, Garvey G (2017). Identifying research priorities to improve cancer control for Indigenous Australians. Public Health Res Pract.

[38] Australian Institute of Health and Welfare (2020) National Cervical Screening Program Monitoring Report 2020, Cancer Series No 130. Cat. No CAN 138. AIHW, Canberra

[39] Dasgupta P, Whop LJ, Diaz A, Cramb SM, Moore SP, Brotherton JM (2019). Spatial variation in cervical cancer screening participation and outcomes among Indigenous and non-Indigenous Australians in Queensland. Geogr Res.

[40] Dasgupta P, Aitken JF, Condon J, Garvey G, Whop LJ, DeBats C (2020). Spatial and temporal variations in cervical cancer screening participation among Indigenous and non-Indigenous women, Queensland, Australia, 2008–2017. Cancer Epidemiol.

[41] Australian Institute of Health and Welfare (2020) BreastScreen Australia monitoring report 2020. Cancer series no. 129. Cat. no. CAN 135.: AIHW, Canberra

[42] Creagh NS, Zammit C, Brotherton JM, Saville M, McDermott T, Nightingale C (2022). The experience of under-screened and never-screened participants using clinician-supported self-collection cervical screening within the Australian national cervical screening program. Women’s Health.

[43] Whop LJ, Butler TL, Lee N, Cunningham J, Garvey G, Anderson K (2022). Aboriginal and Torres Strait Islander women's views of cervical screening by self-collection: a qualitative study. Aust N Z J public Health.

[44] Moore SP, Green AC, Garvey G, Coory MD, Valery PC (2011). A study of head and neck cancer treatment and survival among Indigenous and non-Indigenous people in Queensland, Australia, 1998 to 2004. BMC Cancer.

[45] Howell J, Ward JS, Davies J, Clark PJ, Davis JS (2021). Hepatocellular carcinoma in Indigenous Australians: a call to action. Med J Aust.

[46] Australian Institute of Health and Welfare (2021) Cancer Data in Australia. AIHW, Canberra

[47] Diaz A, Whop LJ, Valery PC, Moore SP, Cunningham J, Garvey G (2015). Cancer outcomes for Aboriginal and Torres Strait Islander Australians in rural and remote areas. Aust J Rural Health.

