# Supplementary material for: Cancer survival differentials for Aboriginal and Torres Strait Islander peoples in Queensland: the impact of remoteness
Source: Cancer Causes Control. 2022 Oct 20;34(1):13–22. doi: 10.1007/s10552-022-01643-1 (PMC9816203; doi:10.1007/s10552-022-01643-1)
Supplement: Supplementary file 1 — Supplementary file1 (DOCX 780 kb) [file 10552_2022_1643_MOESM1_ESM.docx]

SUPPLEMENTARY MATERIAL

**Stata syntax for predictions**

The variable names are as follows:

dxage: Age at diagnosis (integer values between 20 and 89 years)

sage?: Spline terms for age

ra4cat: 4 remoteness areas, coded as 1 to 4.

ethnic: Aboriginal and Torres Strait Islander/Other (coded as 0, 1 respectively)

sex: Male/Female (coded as 1,2, respectively)

e.g. the model for lung cancer:

// The “df’ values entered in the code are as shown in Table 2).

qui rcsgen dxage, gen(sage) df(3) orthog

qui matrix Rage = r(R)

qui global knotsage `r(knots)'

xi: stpm2 sage? sex i.ra4cat ethnic, scale(hazard) df(5) tvc(sage? ra2 ra3 ra4) dftvc(1)

// comparative survival ratio at 1, 5 and 10 years

foreach i in 1 5 10 {

gen t`i' in 1= `i'

}

gen n=0

foreach x in 1 5 10 {

replace n=_n

while n==1 {

predictnl compsur`x'_1 = predict(meansurv timevar(t`x') at(ethnic 0 _Ira4cat_2 0 _Ira4cat_3 0 _Ira4cat_4 0) if2(ethnic==0))/predict(meansurv timevar(t`x') at(ethnic 1 _Ira4cat_2 0 _Ira4cat_3 0 _Ira4cat_4 0) if2(ethnic==0)), ci(cs`x'_1_lci cs`x'_1_uci) force

*In comparison to the same remoteness category

predictnl compsur`x'_2 = predict(meansurv timevar(t`x') at(ethnic 0 _Ira4cat_2 1 _Ira4cat_3 0 _Ira4cat_4 0) if2(ethnic==0))/predict(meansurv timevar(t`x') at(ethnic 1 _Ira4cat_2 1 _Ira4cat_3 0 _Ira4cat_4 0) if2(ethnic==0)), ci(cs`x'_2_lci cs`x'_2_uci) force

predictnl compsur`x'_3 = predict(meansurv timevar(t`x') at(ethnic 0 _Ira4cat_2 0 _Ira4cat_3 1 _Ira4cat_4 0) if2(ethnic==0))/predict(meansurv timevar(t`x') at(ethnic 1 _Ira4cat_2 0 _Ira4cat_3 1 _Ira4cat_4 0) if2(ethnic==0)), ci(cs`x'_3_lci cs`x'_3_uci) force

predictnl compsur`x'_4 = predict(meansurv timevar(t`x') at(ethnic 0 _Ira4cat_2 0 _Ira4cat_3 0 _Ira4cat_4 1) if2(ethnic==0))/predict(meansurv timevar(t`x') at(ethnic 1 _Ira4cat_2 0 _Ira4cat_3 0 _Ira4cat_4 1) if2(ethnic==0)), ci(cs`x'_4_lci cs`x'_4_uci) force

replace n=n+1

}

}

// predicted survival for Aboriginal and Torres Strait Islander & Other

range temptime 0.003 10.2 1010

// Urban

predict survurban_i if ethnic==0, meansurv at(ethnic 0 _Ira4cat_2 0 _Ira4cat_3 0 _Ira4cat_4 0) timevar(temptime) ci

predict survurban_ni if ethnic==0, meansurv at(ethnic 1 _Ira4cat_2 0 _Ira4cat_3 0 _Ira4cat_4 0) timevar(temptime) ci

// Inner regional

predict survinnreg_i if indig==0, meansurv at(indig 0 _Ira4cat_1 1 _Ira4cat_2 0 _Ira4cat_3 0) timevar(temptime) ci

predict survinnreg_ni if ethnic==0, meansurv at(ethnic 1 _Ira4cat_1 1 _Ira4cat_2 0 _Ira4cat_3 0) timevar(temptime) ci

// Outer regional

predict survoutreg_i if ethnic==0, meansurv at(ethnic 0 _Ira4cat_1 0 _Ira4cat_2 1 _Ira4cat_3 0) timevar(temptime) ci

predict survoutreg_ni if ethnic==0, meansurv at(ethnic 1 _Ira4cat_1 0 _Ira4cat_2 1 _Ira4cat_3 0) timevar(temptime) ci

// Remote

predict survremote_i if ethnic==0, meansurv at(ethnic 0 _Ira4cat_2 0 _Ira4cat_3 0 _Ira4cat_4 1) timevar(temptime) ci

predict survremote_ni if ethnic==0, meansurv at(ethnic 1 _Ira4cat_2 0 _Ira4cat_3 0 _Ira4cat_4 1) timevar(temptime) ci

// predicted survival differences for Aboriginal and Torres Strait Islander & Other

* Aboriginal and Torres Strait Islander predictions are first, then subtract Other Qlder predictions

// Urban

predictnl survurbandiff = predict(meansurv at(ethnic 0 _Ira4cat_2 0 _Ira4cat_3 0 _Ira4cat_4 0) timevar(temptime) if2(ethnic==0)) - predict(meansurv at(ethnic 1 _Ira4cat_2 0 _Ira4cat_3 0 _Ira4cat_4 0) timevar(temptime) if2(ethnic==0)), ci(survurbandiff_lci survurbandiff_uci) force

// Inner regional

predictnl survinnregdiff = predict(meansurv at(ethnic 0 _Ira4cat_1 1 _Ira4cat_2 0 _Ira4cat_3 0) timevar(temptime) if2(ethnic==0)) - predict(meansurv at(ethnic 1 _Ira4cat_1 1 _Ira4cat_2 0 _Ira4cat_3 0) timevar(temptime) if2(ethnic==0)), ci(survinnregdiff_lci survinnregdiff_uci) force

// Outer regional

predictnl survoutregdiff = predict(meansurv at(ethnic 0 _Ira4cat_1 0 _Ira4cat_2 1 _Ira4cat_3 0) timevar(temptime) if2(ethnic==0)) - predict(meansurv at(ethnic 1 _Ira4cat_1 0 _Ira4cat_2 1 _Ira4cat_3 0) timevar(temptime) if2(ethnic==0)), ci(survoutregdiff_lci survoutregdiff_uci) force

// Remote

predictnl survremotediff = predict(meansurv at(ethnic 0 _Ira4cat_2 0 _Ira4cat_3 0 _Ira4cat_4 1) timevar(temptime) if2(ethnic==0)) - predict(meansurv at(ethnic 1 _Ira4cat_2 0 _Ira4cat_3 0 _Ira4cat_4 1) timevar(temptime) if2(ethnic==0)), ci(survremotediff_lci survremotediff_uci) force

Supplementary Table S1. Number of diagnosed cancers and population by ethnicity and remoteness categories, Queensland, 1997-2016

|  | Aboriginal and Torres Strait Islanders | | | | Other Queenslanders | | | |
| --- | --- | --- | --- | --- | --- | --- | --- | --- |
| Type of cancer | Urban | Inner regional | Outer regional | Remote | Urban | Inner regional | Outer regional | Remote |
| *All cancers* | 1,664 | 1,048 | 1,864 | 1,215 | 223,776 | 85,797 | 51,323 | 7,193 |
| Head and neck cancers | 69 | 46 | 130 | 111 | 5,859 | 2,344 | 1,827 | 289 |
| Oesophageal cancer | 27 | 18 | 58 | 33 | 2,220 | 876 | 608 | 82 |
| Stomach cancer | 26 | 24 | 37 | 26 | 3,547 | 1,270 | 742 | 85 |
| Colorectal cancer | 170 | 91 | 160 | 93 | 27,549 | 10,819 | 6,429 | 890 |
| Liver cancer | 33 | 12 | 44 | 48 | 2,132 | 696 | 495 | 59 |
| Pancreatic cancer | 37 | 28 | 50 | 22 | 4,320 | 1,676 | 901 | 139 |
| Lung cancer | 208 | 154 | 297 | 193 | 18,546 | 7,306 | 4,530 | 756 |
| Breast cancer | 226 | 148 | 231 | 129 | 30,871 | 10,506 | 6,143 | 786 |
| Cervical cancer | 39 | 39 | 79 | 46 | 1,935 | 590 | 447 | 72 |
| Prostate cancer | 165 | 122 | 154 | 77 | 32,540 | 14,283 | 8,227 | 1,173 |
| Leukaemia | 41 | 27 | 39 | 22 | 5,929 | 2,327 | 1,385 | 181 |
| Population (2015 only) | 68,098 | 42,600 | 63,355 | 33,973 | 2,925,039 | 923,085 | 632,821 | 89,883 |

Population numbers by SA2 and ethnicity sourced from Queensland Government Statistician’s Office, Queensland Treasury (<https://www.qgso.qld.gov.au/statistics/theme/population/aboriginal-peoples-torres-strait-islander-peoples/population-estimates-projections>) and are based on the 2011 Australian Statistical Geography Standard for consistency with the cancer data.

Supplementary Table S2. Number of diagnosed cancers and population by ethnicity and socioeconomic categories, Queensland, 1997-2016

|  | Aboriginal and Torres Strait Islanders | | | Other Queenslanders | | |
| --- | --- | --- | --- | --- | --- | --- |
| Type of cancer | Advantaged | Average | Disadvantaged | Advantaged | Average | Disadvantaged |
| *All cancers* | 1,664 | 1,048 | 1,864 | 223,776 | 85,797 | 51,323 |
| Head and neck cancers | 69 | 46 | 130 | 5,859 | 2,344 | 1,827 |
| Oesophageal cancer | 27 | 18 | 58 | 2,220 | 876 | 608 |
| Stomach cancer | 26 | 24 | 37 | 3,547 | 1,270 | 742 |
| Colorectal cancer | 170 | 91 | 160 | 27,549 | 10,819 | 6,429 |
| Liver cancer | 33 | 12 | 44 | 2,132 | 696 | 495 |
| Pancreatic cancer | 37 | 28 | 50 | 4,320 | 1,676 | 901 |
| Lung cancer | 208 | 154 | 297 | 18,546 | 7,306 | 4,530 |
| Breast cancer | 226 | 148 | 231 | 30,871 | 10,506 | 6,143 |
| Cervical cancer | 39 | 39 | 79 | 1,935 | 590 | 447 |
| Prostate cancer | 165 | 122 | 154 | 32,540 | 14,283 | 8,227 |
| Leukaemia | 41 | 27 | 39 | 5,929 | 2,327 | 1,385 |
| Population (2015 only) | 12,980 | 111,273 | 83,773 | 934,765 | 2,861,912 | 774,092 |

Population numbers by SA2 and ethnicity sourced from Queensland Government Statistician’s Office, Queensland Treasury (<https://www.qgso.qld.gov.au/statistics/theme/population/aboriginal-peoples-torres-strait-islander-peoples/population-estimates-projections>) and are based on the 2011 Australian Statistical Geography Standard for consistency with the cancer data.

Supplementary Table S3. Standardised 10-year cause-specific survival estimates by ethnicity and remoteness, Queensland, 1997-2016

|  | 10-year standardised survival (95% CI) | | | |
| --- | --- | --- | --- | --- |
| Cancer | **Urban** | | **Remote** | |
|  | **First Nations** | **Other Queenslanders** | **First Nations** | **Other Queenslanders** |
| *All cancers* | *76.5 (75.1, 77.9)* | *80.2 (80.0, 80.4)* | *60.0 (58.3, 61.9)* | *64.9 (64.7, 65.1)* |
| Head and neck cancers | 75.7 (72.3, 79.3) | 87.1 (86.2, 88.0) | 50.5 (45.5, 56.1) | 70.4 (68.8, 72.1) |
| Oesophageal cancer | 41.9 (27.8, 63.1) | 57.3 (54.8, 60.0) | 16.5 (7.1, 38.5) | 31.6 (28.9, 34.6) |
| Stomach cancer | 46.4 (39.1, 55.1) | 57.6 (55.7, 59.5) | 22.5 (16.0, 31.7) | 34.2 (31.7, 37.0) |
| Colorectal cancer | 89.4 (86.4, 92.6) | 88.6 (88.2, 88.9) | 73.8 (67.1, 81.1) | 71.9 (71.2, 72.6) |
| Liver cancer | 44.4 (37.4, 52.7) | 50.6 (48.3, 53.1) | 19.3 (13.7, 27.1) | 25.0 (22.8, 27.4) |
| Pancreatic cancer | 32.7 (22.6, 47.1) | 37.2 (35.6, 38.9) | 11.4 (5.8, 22.6) | 14.6 (13.3, 16.0) |
| Lung cancer | 44.0 (41.2, 47.0) | 49.6 (48.7, 50.5) | 18.3 (15.9, 21.0) | 23.3 (22.3, 24.4) |
| Breast cancer | 96.8 (96.3, 97.5) | 98.1 (98.0, 98.3) | 85.3 (82.9, 87.8) | 91.0 (90.6, 91.5) |
| Cervical cancer | 81.8 (77.5, 86.4) | 90.2 (89.0, 91.5) | 61.0 (53.9, 69.0) | 77.0 (74.8, 79.3) |
| Prostate cancer | 96.7 (96.0, 97.5) | 96.7 (96.0, 97.5) | 87.3 (84.7, 89.9) | 87.3 (84.7, 89.9) |
| Leukaemia | 84.5 (77.0, 92.8) | 86.7 (85.8, 87.6) | 71.0 (58.8, 85.6) | 74.8 (73.4, 76.2) |

Supplementary Figure S1. Standardised survival estimates for First Nations peoples and other Queenslanders by remoteness category, 1997-2016


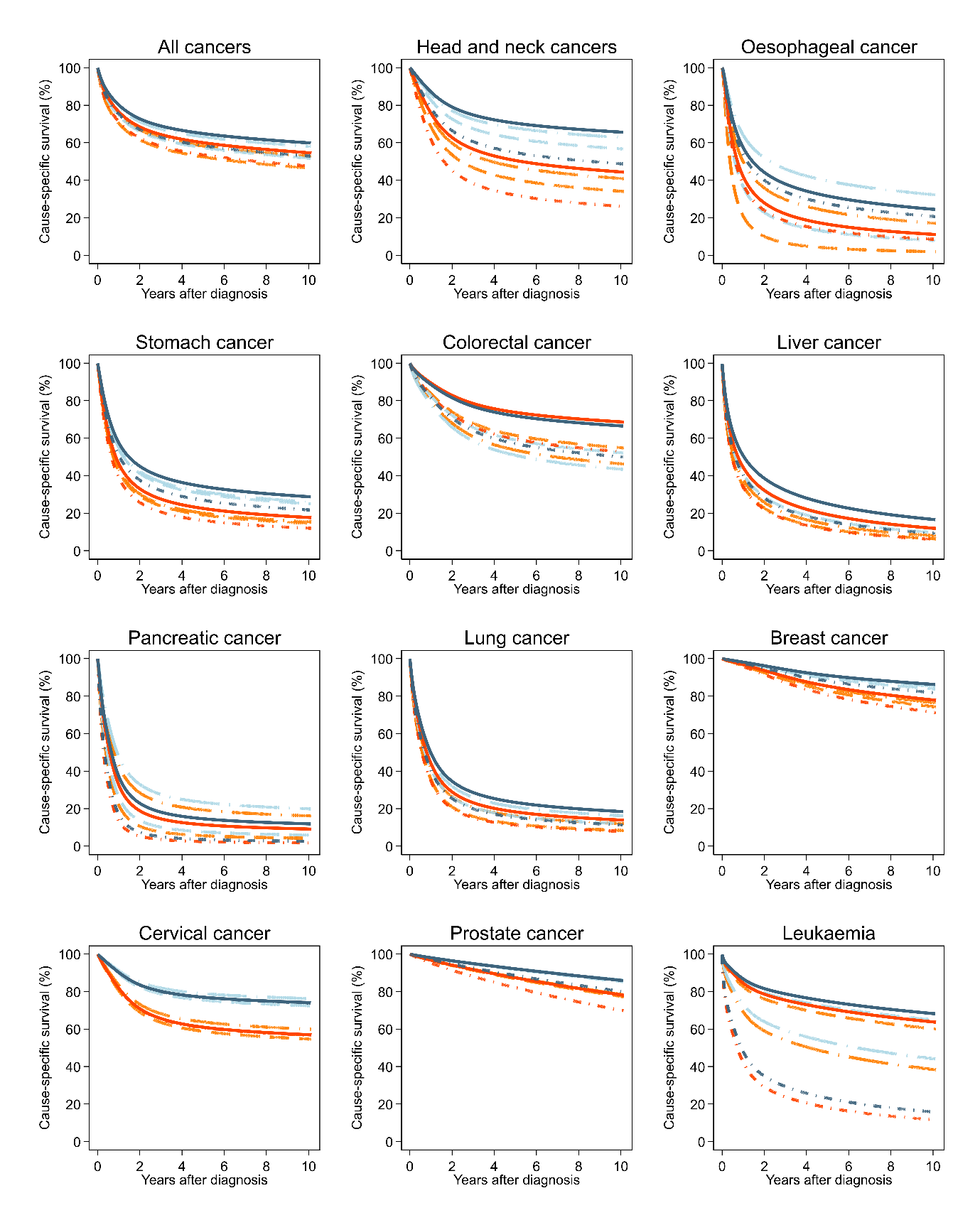

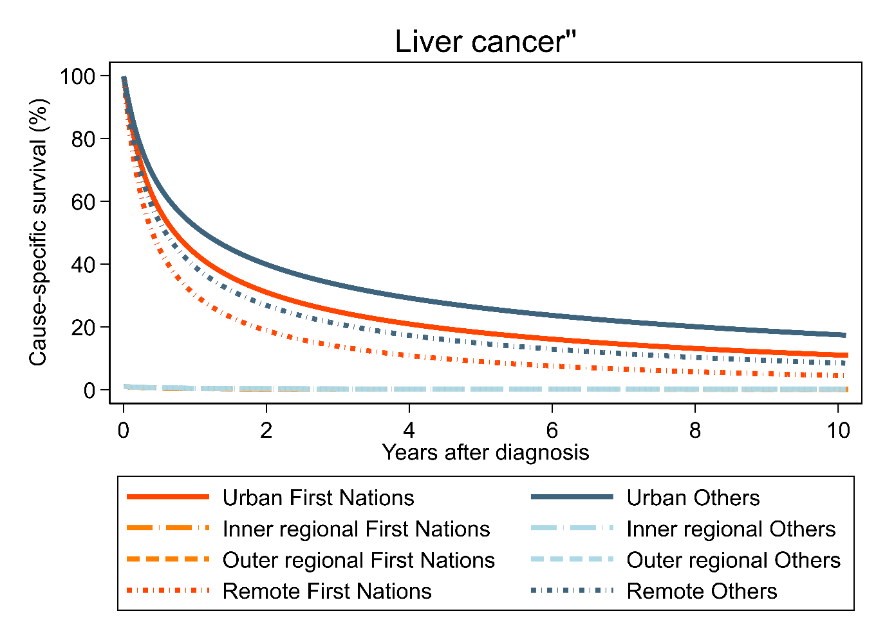


Supplementary Figure S2. Standardised survival differences between First Nations peoples and other Queenslanders by remoteness category, 1997-2016


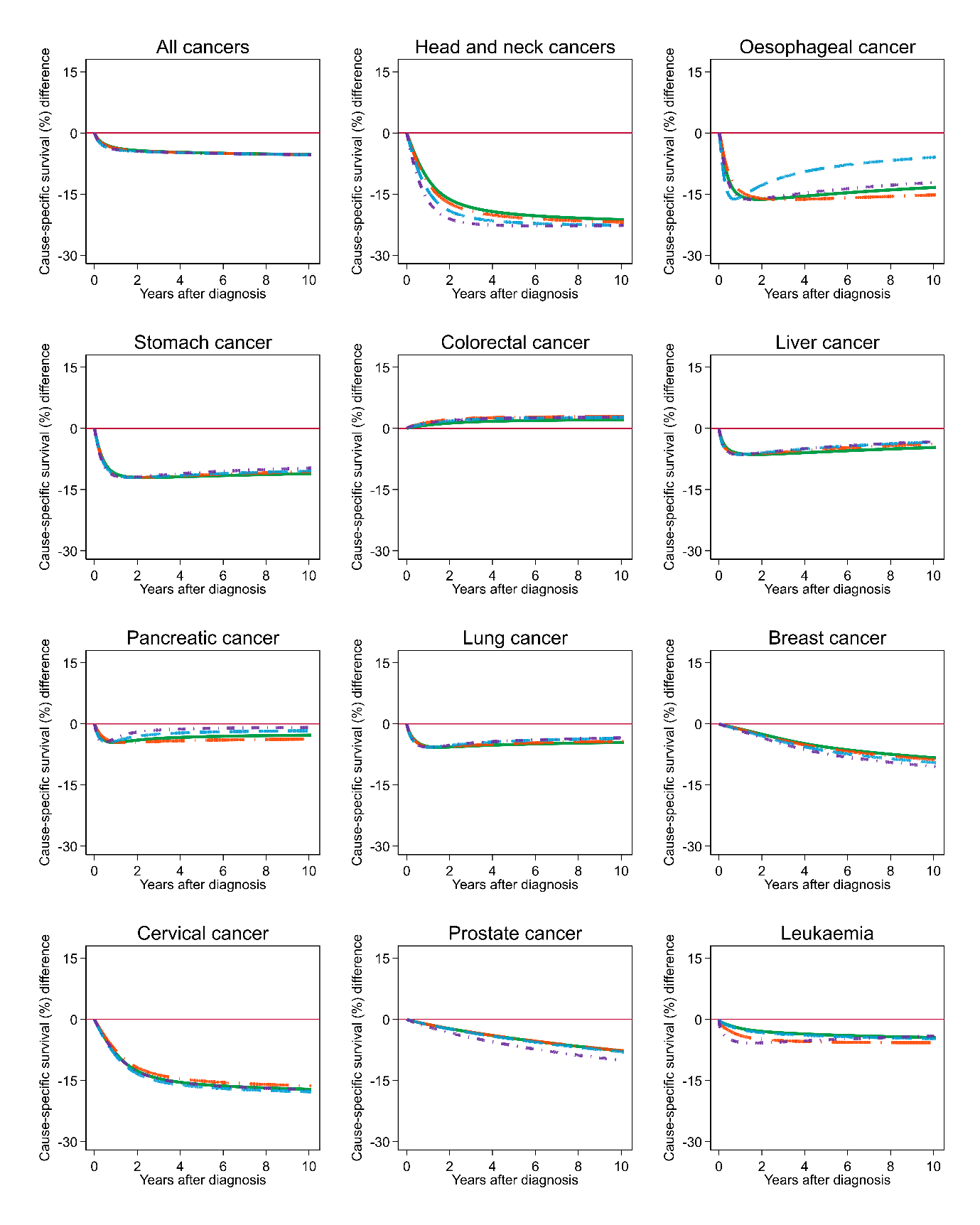


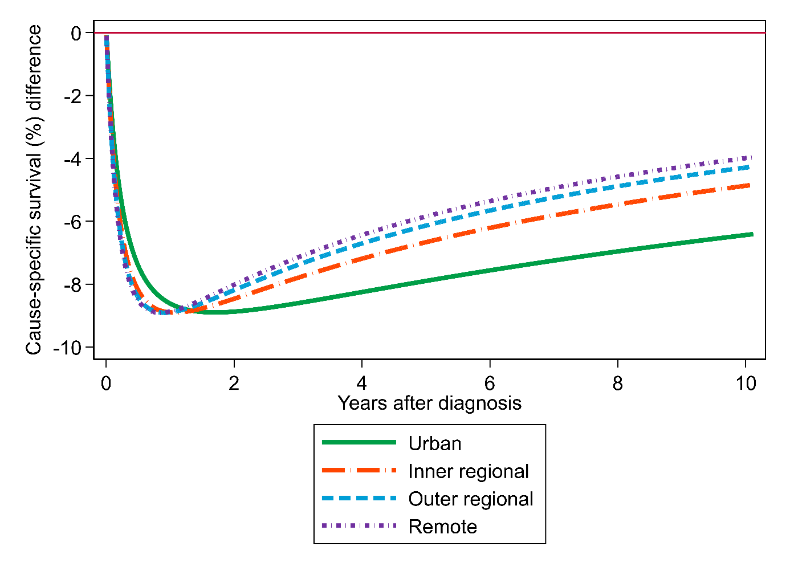


Supplementary Figure S3. Ten-year comparative survival ratios by cancer type and remoteness, Queensland, 1997-2016.


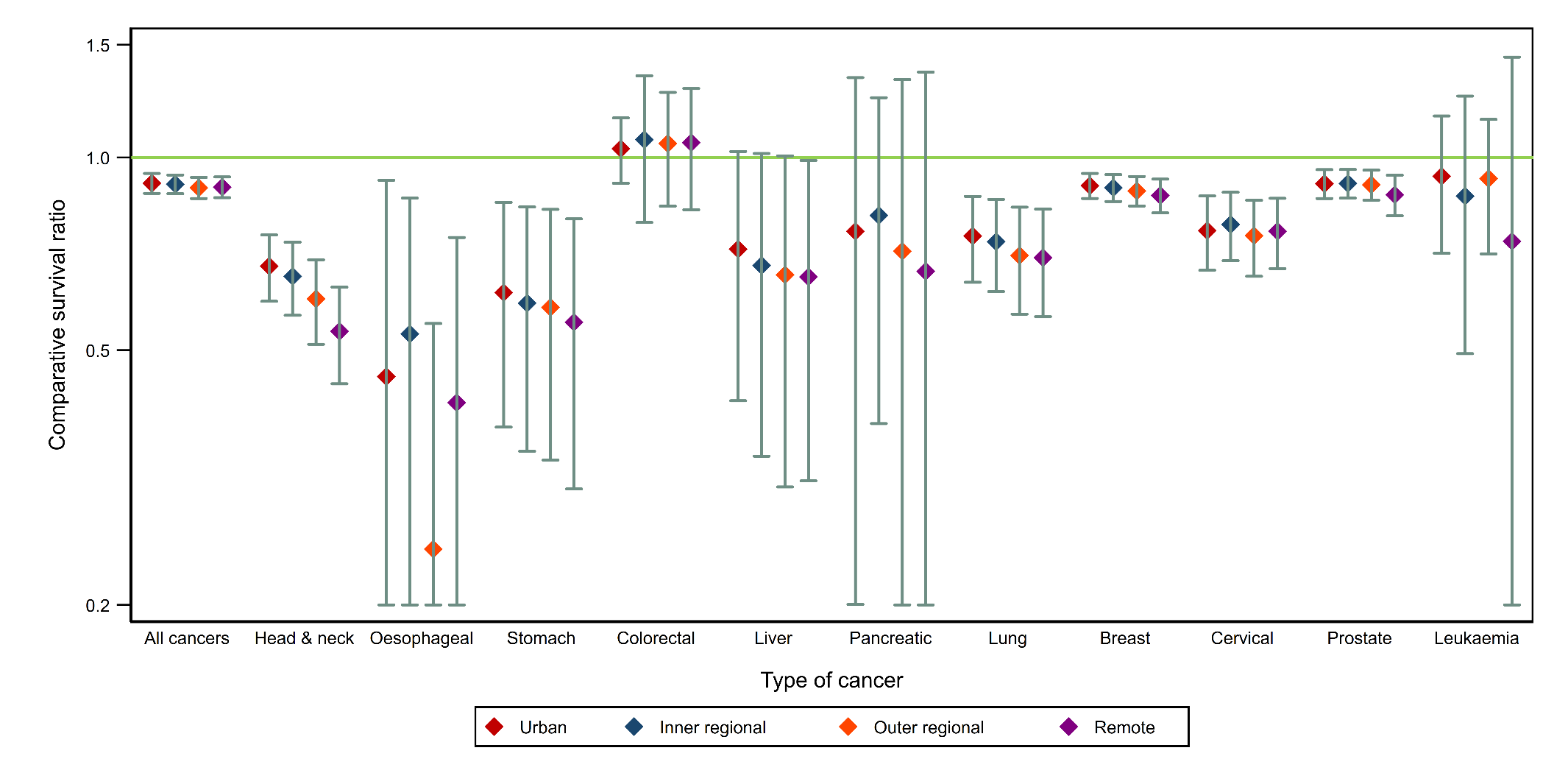


Notes: The comparative survival ratio is the standardised cause-specific 10-year survival for First Nations people divided by other Queenslanders. A value of 1 means First Nations survival up to 10 years is equivalent to other Queenslander survival.

Capped lines show the 95% confidence interval, and have been truncated to 0.2 if they extended beyond this.

Urban is the Major City remoteness area. Remote is the combined Remote and Very Remote remoteness areas.

Breast cancer is for females only.
